# Dominant trajectory as a longitudinal framework for multiple ground-glass nodules

**DOI:** 10.3389/fmed.2026.1979153

**Published:** 2026-10-22

**Authors:** Huijuan Cheng, Zhe Zhang, Yanyan Ye, Feng Lu

**Affiliations:** 1 Department of Respiratory Medicine, The Second Affiliated Hospital of Fujian University of Traditional Chinese Medicine, Fuzhou, China; 2 Fujian University of Traditional Chinese Medicine, Fuzhou, China

**Keywords:** dominant trajectory, longitudinal risk prioritization, lung cancer risk, lung preservation, multiple ground-glass nodules, subsolid nodules

## Introduction

Multiple pulmonary ground-glass nodules (GGNs) require decisions about both individual lesions and the lungs as a whole. Here, GGNs encompass pure ground-glass and part-solid nodules; ground-glass opacity (GGO) describes the imaging appearance. Existing guidelines already incorporate serial change, solid-component size, morphology, and patient fitness (1–3). The 2023 American Association for Thoracic Surgery (AATS) consensus specifically addresses multiple subsolid nodules, and the European Association for Cardio-Thoracic Surgery/European Society of Thoracic Surgeons guidance integrates lesion evolution with operative suitability (2, 3). A Chinese multidisciplinary consensus also addresses multiple GGN-like lung cancers (4). Longitudinal assessment is therefore already central to the management of multiple GGNs.

In this narrative Opinion, “dominant trajectory” describes how clinical priority among nodules evolves over time. Lesion-specific imaging findings are considered alongside the status of the other nodules, treatment capacity, and patient goals. The trajectory concerns decisions across visits rather than the biological natural history of a single lesion. Recording why priority is maintained or changed may improve continuity of multidisciplinary care. The concept remains unvalidated and provides neither a malignancy probability nor an independent indication for treatment; a GGN itself does not establish cancer.

## Evidence supporting reassessment and its limits

The available outcome evidence supports control of the main cancer. In Shimada et al.'s surgical series of 67 patients, 39 had 118 unresected GGOs; 8% of residual nodules grew, and new GGOs appeared in 15 patients (23%). Survival was associated with main-cancer characteristics, whereas residual growth or new lesions were not associated with survival (5). Bongiolatti et al. similarly reported favorable outcomes after dominant-lesion surgery with observation and selective treatment of other lesions (6). These selected observational cohorts support current management; they do not establish that re-ranking lesions improves survival or that residual growth itself worsens prognosis.

Natural-history evidence nevertheless shows why serial characterization matters. In a prospective multicenter study of 795 patients with 1,229 subsolid nodules, 56 of 1,046 initially pure GGNs (5.4%) became part-solid during mean follow-up of 4.3 years (7). These observations make periodic reassessment clinically relevant. Whether a structured record of changing priority improves decisions or outcomes beyond guideline-concordant care remains to be tested.

Lesion-specific evidence also depends on nodule type. Development of a solid component in a pure GGN, or enlargement of an existing component in a part-solid nodule, warrants particular attention. Changes in the diameter or attenuation of a persistent pure GGN require interpretation alongside morphology and CT comparability (2, 3). In the multicenter retrospective GORDON study, Catelli et al. found that larger preoperative diameter and higher CT attenuation were associated with invasive histology in resected pure ground-glass adenocarcinomas (8). The association supports attention to features beyond size alone. Because the study assessed preoperative images in a selected surgical cohort, its findings do not define thresholds for intervention or the meaning of serial attenuation changes.

## What the framework adds to existing guidance

Clinical teams already reassess the most concerning nodule over time. A shared record can carry the reasoning from one review to the next: which lesion or lesions now take priority and why, what has changed or remained stable, how certain the imaging assessment is, and the status of the other nodules. When an intervention is considered, the record should also describe how it may affect future treatment options. Priority may change even if management does not. Documenting these decisions consistently across visits and clinicians may support continuity and allow its practical value to be evaluated.

Figure 1 illustrates how the four assessment domains—lesion evidence, remaining nodules, future capacity, and patient priorities—inform a change in priority. The larger pure GGN remains stable while the smaller lesion develops an enlarging solid component. In that setting, attention may shift toward the smaller lesion after considering imaging reliability and the options available for other nodules. Two or more lesions may be co-dominant when they raise similar concern, warranting parallel assessment. New diffuse or rapidly changing opacities warrant consideration of inflammation or other diagnoses. The framework keeps these judgments within established diagnostic and treatment criteria.

**Figure 1:**
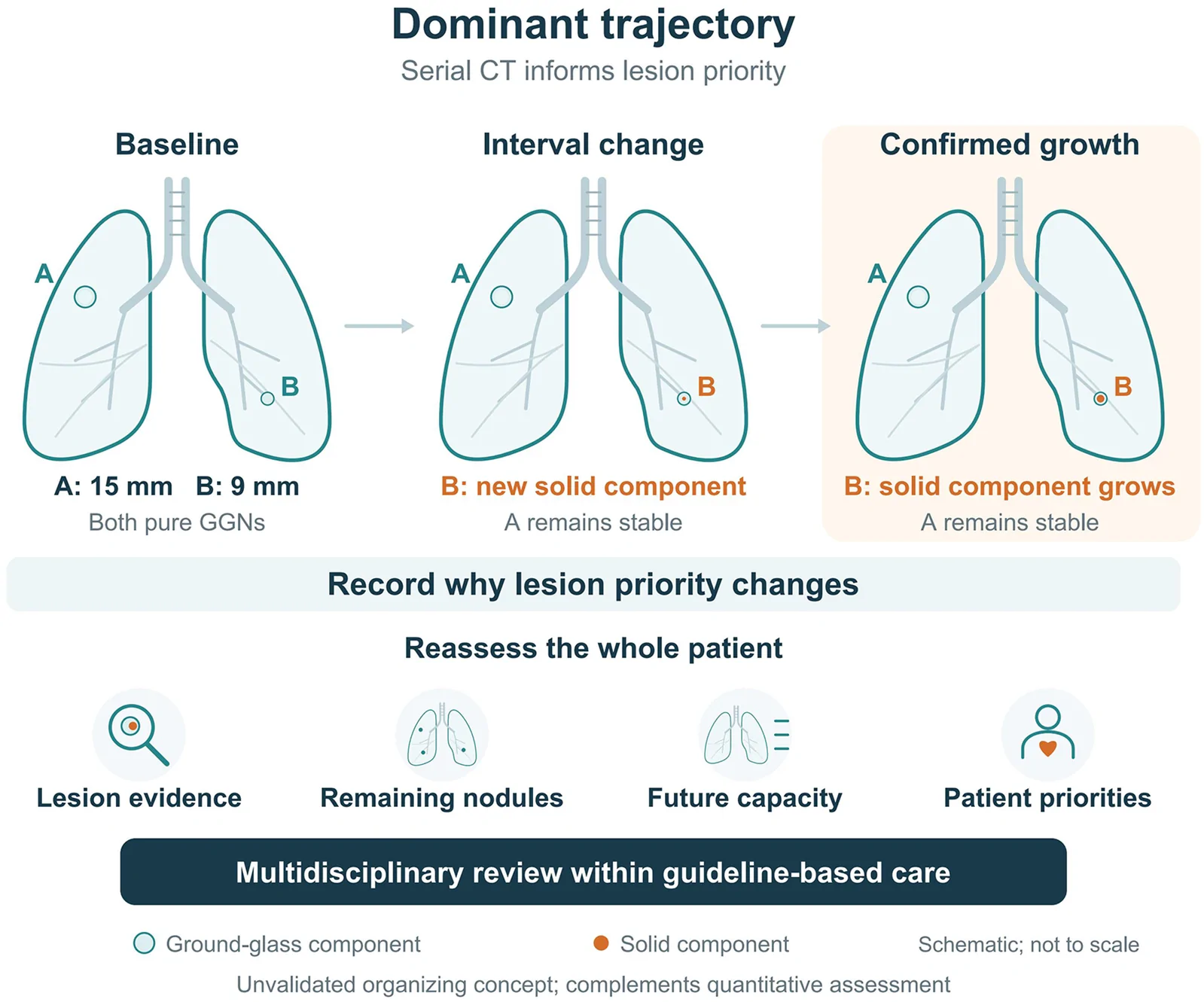
Dominant-trajectory framework for multiple ground-glass nodules (GGNs). In this hypothetical sequence, lesion A remains a stable pure GGN while lesion B develops an enlarging solid component, shifting clinical attention toward B. Reassessment considers lesion evidence, the remaining nodules, future treatment capacity, and patient priorities. Similarly concerning lesions may be co-dominant and warrant parallel assessment. Pale teal represents ground-glass tissue and orange represents solid tissue. Sizes and anatomy are schematic and not to scale; the observations do not specify follow-up intervals.

## Relationship to quantitative growth assessment

Dominant trajectory complements volume doubling time (VDT) and semi-automated volumetry; it does not replace them. The British Thoracic Society guideline already incorporates quantitative growth assessment and emphasizes measurement quality (9). When serial segmentation is reliable, lesion volume, VDT, and, where appropriate, mass change can inform prioritization. Song et al. reported median VDTs of approximately 1,832, 1,229, and 759 days among volume-growing subsolid nodules categorized as pure GGNs, part-solid nodules with solid components ≤5 mm, and those with solid components >5 mm, respectively (10). These observational values are not intervention cutoffs, and VDT thresholds derived mainly from solid nodules should not be transferred uncritically to GGNs.

Technical uncertainty must remain visible. In a same-day repeat-CT study of 69 subsolid nodules, longest-diameter variability was ±1.3 mm with the same reader and ±2.2 mm with different readers; solid-component measurements were particularly variable between readers (11). This supports checking acquisition, reconstruction, segmentation, and reader consistency before interpreting small changes. Increasing solid tissue can matter despite little change in overall diameter or volume. A long estimated VDT should therefore not overrule convincing solid-component evolution. The framework brings these measurements and their uncertainty into the patient-level priority record.

## An illustrative decision and surveillance burden

Consider a hypothetical patient with a 15-mm pure GGN (lesion A) and a separate 9-mm pure GGN (lesion B), as illustrated in Figure 1. On technically comparable follow-up examinations, A remains unchanged while B develops a solid component that reproducibly enlarges on subsequent imaging. Guideline-based assessment may therefore prioritize B despite its smaller overall size. A dominant-trajectory record would state why priority changed, document the imaging uncertainty, and consider the pulmonary reserve and anatomy needed for later treatment of other nodules. Multidisciplinary review may support surveillance, further evaluation, or treatment according to established criteria. If treatment is indicated, the remaining lesions can be reassessed afterward. The illustrated stages show observations rather than a prescribed imaging schedule.

Reassessment need not mean additional CT examinations. For eligible adults with incidentally detected multiple subsolid nodules, Fleischner guidance recommends initial CT at 3–6 months, with subsequent management determined by persistence, size, and the most suspicious nodule. Selected persistent subsolid nodules warrant surveillance extending to 5 years (1). These recommendations are not screening protocols and do not directly govern patients with known cancer. After lung-cancer treatment, surveillance should follow the relevant oncologic and residual-nodule context. A new label should not automatically shorten intervals or justify indefinite imaging.

Low-dose thin-section CT and coordination of examinations can reduce unnecessary exposure (9). Follow-up intensity and duration should reflect lesion evolution, life expectancy, competing illness, ability to undergo treatment, and informed preferences. Cumulative radiation, visits, incidental findings, and financial burden belong in the decision, alongside the risk of missing meaningful progression. The framework should make these tradeoffs explicit rather than assume that more surveillance is always better.

## Preserving treatment capacity and addressing patient priorities

Preserving lung tissue is relevant when several interventions may be needed, but requires oncologically appropriate selection. JCOG0802/WJOG4607L randomized 1,106 patients with peripheral non-small-cell lung cancer ≤2 cm and consolidation-to-tumor ratio >0.5. Five-year overall survival was 94.3% after segmentectomy versus 91.1% after lobectomy, but local relapse was more frequent (10.5% versus 5.4%); the 1-year difference in median forced expiratory volume in 1 s decline was only 3.5 percentage points (12). CALGB 140503 randomized 697 patients with peripheral tumors ≤2 cm and intraoperatively confirmed node-negative disease; sublobar resection was noninferior for disease-free survival (hazard ratio 1.01; 90% confidence interval 0.83–1.24) (13). Oncologically appropriate parenchymal-sparing surgery may preserve options for treating other nodules later, making future treatment capacity relevant to the current surgical decision. These trials do not establish that repeated sublobar resections are advantageous in multifocal GGN disease or that large functional benefits should be expected.

For patients with a treatment-requiring lesion who are unsuitable for surgery, stereotactic body radiotherapy or thermal ablation may be considered after multidisciplinary review (2, 9). Their potential to avoid anatomical resection must be weighed against location, diagnostic certainty, local control, toxicity, and feasibility of retreatment. Neither modality should be used merely to clear stable GGNs. The dominant-trajectory proposal cannot establish their equivalence to surgery or specify an optimal sequence of local therapies.

Patient anxiety should be addressed through clear explanations of uncertainty, a written follow-up plan, and access to support. Yuan et al.'s nonrandomized cohort of 200 patients with multiple pulmonary nodules associated multidisciplinary care with better health knowledge, coping, quality of life, and satisfaction at 6 months (14). It did not establish improved cancer detection or oncologic outcomes, and was not restricted to multifocal GGNs. This experience reinforces the value of support during surveillance. Preferences can inform acceptable surveillance and treatment burdens; anxiety alone should not be interpreted as biological progression.

## Discussion

The proposed framework makes lesion hierarchy and its rationale explicit while retaining established diagnostic and treatment criteria. Its strongest justification is practical clarity; its central limitation is the absence of demonstrated incremental clinical benefit. Qualitative prioritization may vary between clinicians, amplify measurement noise, increase workload, or encourage unnecessary intervention if “trajectory” is mistaken for a validated risk estimate. Evidence drawn from selected surgical cohorts and individual-nodule studies also limits generalizability to the full population with multiple GGNs.

Prospective evaluation should compare structured documentation with usual guideline-concordant multidisciplinary care, using common imaging and intervention criteria. Initial outcomes should include interobserver agreement on the priority lesion, reproducibility of a documented priority change, decision concordance, and additional workload. Patient-level outcomes should include stage at intervention, cancer-specific survival, pulmonary function, cumulative procedures, treatment morbidity, CT exposure, anxiety, and quality of life. Analyses must account for baseline nodule burden, prior cancer, comorbidity, and competing mortality. Improved documentation alone would not establish a survival advantage or justify delaying indicated treatment.

Dominant trajectory should therefore remain a hypothesis-generating framework for longitudinal care. Its value will depend on whether explicit documentation improves decisions and patient outcomes beyond what current guidance already achieves, while preserving appropriate treatment of the lesion that matters now.
